# Supplementary material for: Decline of adolescent smoking in Ireland 1995–2015: trend analysis and associated factors
Source: BMJ Open. 2018 Apr 27;8(4):e020708. doi: 10.1136/bmjopen-2017-020708 (PMC5922513; doi:10.1136/bmjopen-2017-020708)
Supplement: Supplementary data [file bmjopen-2017-020708supp001.pdf]

Appendix I: Real price per package of 20 cigarettes in Ireland, 1995-2015

Year	1995	1996	1997	1998	1999	2000	2001	2002	2003	2004	2005	2006	2007	2008	2009	2010	2011	2012	2013	2014	2015
Retail price	3.5	3.6	3.8	3.9	4.1	4.8	4.9	5.2	5.8	6.2	6.3	6.4	7.0	7.9	8.4	8.5	8.6	9.1	9.5	10.0	10.5
CPI (year 1995=100)	100	102	103	106	107	113	119	124	129	131	135	140	147	153	146	145	148	151	152	152	151
Real price(base year=1995)	3.5	3.6	3.6	3.7	3.8	4.2	4.1	4.2	4.5	4.7	4.6	4.6	4.8	5.2	5.7	5.8	5.8	6.0	6.2	6.6	6.9
Real price change (%)		2.6	1.9	2.4	1.6	11.6	-2.7	1.9	8.5	3.2	-1.0	-1.5	4.6	7.8	11.0	2.2	-1.4	4.9	3.3	5.3	5.3

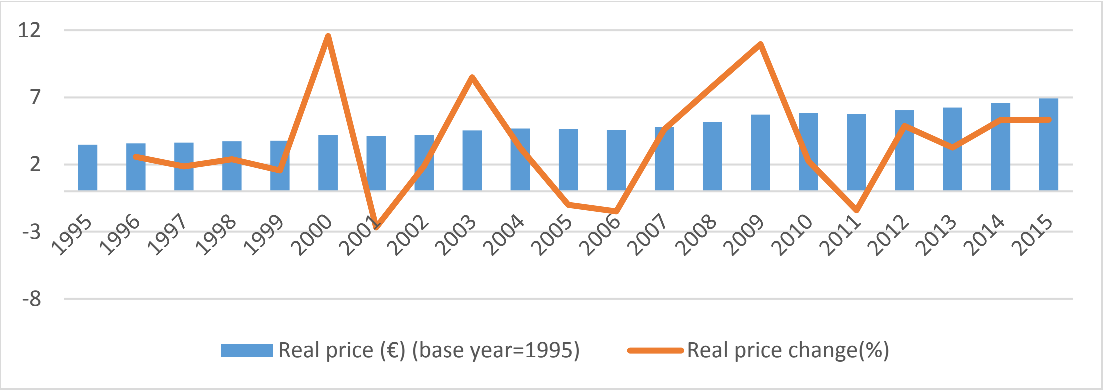

Appendix II: Timestamps of Tobacco Control Policies in Ireland, 1995-2015

2000
- Legislation extended the advertising ban to include print media and some forms of sponsorship.

2001
- NRT is available on prescription free of charge to medical card holders (not all polulation).

2002
- Legislation banned some forms of indirect marketing, such as mail giveaways, promotional discounts and sponsored events.
- Ireland has rotating warnings which cover 30% of the package in front and 40% of the package on the back (in accordance with EU requirements).
- Under the Public Health (Tobacco) Act 2002 it is an offence to sell cigarettes or other tobacco products to anyone under the age of 18. (Not implemented till 2007)

2003
- Ireland introduced Quiline.

2004
- Smoke-free law came into effect, which applies to all worksites, including bars and restaurants.
- Bans in advertising were extended to all forms of direct advertising in major media including billboards and more indirect advertising (ban on packages less than 20 sticks, sponsorship misleading false packaging)

2007
- Since 2 April, it is an offence to sell cigarettes or other tobacco products to persons aged under 18 years.
- A ban on packets containing less than 20 cigarettes and the sale of confectionaries that resemble cigarettes.

2008
- All products going to market after October 2008 are required to carry health warnings in both English and Irish and thus the warning size was increased to 32% of the front of the package and 45% of the rear of the package in accordance with EC Directive EC/37/2001.

2009
- Ban on point of sale display and advertising of tobacco products.
- Self-service vending machines are prohibited except in licensed premises and registered clubs and must be operated in accordance with Regulations.

2013
- Graphic warnings must be placed on any tobacco product on the market.

2014
- Since July 2014, HPRA has announced that NRT have been authorised for sale in general retail and grocery outlets, no prescription needed.
