# Supplementary material for: Decline of adolescent smoking in Ireland 1995–2015: trend analysis and associated factors
Source: BMJ Open. 2018 Apr 27;8(4):e020708. doi: 10.1136/bmjopen-2017-020708 (PMC5922513; doi:10.1136/bmjopen-2017-020708)
Supplement: Author's manuscript [file bmjopen-2017-020708.draft_revisions.pdf]

BMJ Open

BMJ Open is committed to open peer review. As part of this commitment we make the peer review history of every article we publish publicly available.

When an article is published we post the peer reviewers' comments and the authors' responses online. We also post the versions of the paper that were used during peer review. These are the versions that the peer review comments apply to.

The versions of the paper that follow are the versions that were submitted during the peer review process. They are not the versions of record or the final published versions. They should not be cited or distributed as the published version of this manuscript.

BMJ Open is an open access journal and the full, final, typeset and author-corrected version of record of the manuscript is available on our site with no access controls, subscription charges or pay-per-view fees (http://bmjopen.bmj.com).

If you have any questions on BMJ Open's open peer review process please email info.bmjopen@bmj.com

BMJ Open

Decline of Adolescent Smoking in Ireland 1995-2015: Trend Analysis and Associated Factors

Journal:	*BMJ Open*
Manuscript ID	bmjopen-2017-020708
Article Type:	Research
Date Submitted by the Author:	17-Nov-2017

Complete List of Authors:	Li, Shasha; TobaccoFree Research Institute Ireland,<br>Keogan, Sheila; TobaccoFree Research Institute Ireland<br>Taylor, Keishia; TobaccoFree Research Institute Ireland<br>Clancy, Luke; TobaccoFree Research Institute Ireland Dublin, IE

Keywords:	PUBLIC HEALTH, EPIDEMIOLOGY, PREVENTIVE MEDICINE

SCHOLARONE™
Manuscripts

For peer review only

Title: Decline of Adolescent Smoking in Ireland 1995-2015: Trend Analysis and Associated Factors

Corresponding Author: Shasha Li

Address for all authors: TobaccoFree Research Institute Ireland,

Focas Research Institute,

DIT, Kevin Street,

Dublin 8, Ireland

Email: shashali@tri.ie

Tel: +35312601966

Co-authors:

1. Sheila Keogan
2. Keishia Taylor
3. Luke Clancy

Word Count: 3257

For peer review only

ABSTRACT

Objectives

The study examines trends in smoking among Irish adolescents of 15-16 years old between 1995 and 2015 and the factors associated with their smoking behaviours between 2007 and 2015.

Methods

Data were obtained from the European School Survey Project on Alcohol and Other Drugs (ESPAD) Ireland between 1995 and 2015. To examine the gender gap, two-sample proportion tests were used. Multivariate logistic regression was performed to examine the factors associated with smoking behaviours. Dependent variable is whether a respondent is a smoker in last-30 day. Independent variables includes gender, survey years, perceived ease of access to cigarettes, perceived risk of smoking, perceived relative wealth, parental monitoring, maternal relationship, family structure , truancy and peer smoking.

Results

Smoking prevalence has dropped from 41% in 1995 to 13% in 2015. The prevalence was much higher among girls than boys in 1995. The gender gap was closed by 2015. Multivariate regression results show that peer smoking, perceived access to cigarettes, perceived risks of smoking, parental monitoring, truancy, maternal relationship, perceived relative wealth and family structure were all significantly associated with adolescent smoking, and some of the factors had different effects for female and male students.

Conclusion

Ireland has successfully achieved a considerable decrease of adolescent smoking from 1995 to 2015, during which various tobacco control policies have been implemented. In addition,

For peer review only

the gender gap on adolescent smoking has been closed during the period. Adolescent smoking could be further improved through strengthening enforcement on adolescent access to cigarettes and maintaining a high-intensity tobacco control media campaign targeting adolescent. Parents could also contribute by enhancing monitoring.

Keywords: Public health; Epidemiology; Preventive medicine

For peer review only

Strengths and limitations of this study

- The data is from the best available survey on Irish adolescents from 1995-2015 using an internationally validated survey instrument, including comprehensive measurement for smoking-related factors. The data sets in different years are comparable in data collection and questions.
- The study is based on multivariable logistic regression, which controls for confounders.
- The factors associated with the changing gender gap in adolescent smoking was examined.
- Other factors potentially related to adolescent smoking were not included in the surveys, such as parental smoking.
- Limited data availability for some of the ESPAD waves are acknowledged.

For peer review only

INTRODUCTION

People who take up smoking at a younger age become more dependent and find it harder to quit than smokers who start later in their lives,[1 2] so policies designed to discourage adolescent from starting to smoke have been at the forefront of tobacco prevention in recent years. In Ireland, the Tobacco Free Ireland (TFI) report of 2013 stated that the protection of children must be prioritised in all of the initiatives outlined in the policy.[3]

The parties to the 2003 WHO Framework Convention on Tobacco Control stated their "deep concern" regarding tobacco consumption by children and adolescents, emphasising price and tax measures as an effective means of reducing tobacco consumption among young people.[4] Between 1995 and 2015, the real retail price per package of 20 cigarettes in Ireland has been increased almost every year (See Appendix I). The European Tobacco Products Directive (2014/40/EU) places restrictions on the use of tobacco packaging designed to appeal to children and prohibits flavoured cigarettes and roll-your-own tobacco.[5] In Ireland, stronger legislation regarding the complete standardisation of tobacco packaging came into force in September 2017 and should reduce further the effect of tobacco advertising on adolescents.[6]

Between 1995 and 2015, a series of national level tobacco control policies were introduced (see Appendix II), although there were no school-specific tobacco control policies implemented in Ireland. For example, the implementation of age limit law under Public Health (Tobacco) Act 2002 was not fully implemented until April 2007. It is an offence to sell cigarettes or other tobacco products to persons aged under 18 years. In addition, since 2009 retailers are required to register with the National Tobacco Control Office. Vending machines were banned except in licensed establishments.

For peer review only

There has been a large volume of research conducted regarding interventions against adolescent smoking, including studies evaluating policies to restrict access and raise awareness of risk, with mixed results.[7 8 9] Some studies have examined perceptions of risk and its association with smoking and the majority reported a negative association between risk perception and smoking.[10 11] Other studies have investigated correlates in the domestic and social sphere, including associations with parental monitoring, relationships with parents, family structure, truancy from school, and peer smoking. [12 13 14 15 16] It is also established that adolescents from a lower socioeconomic background are more likely to smoke.[17 18] This study first establishes the declining trends in smoking among Irish 15 and 16 year olds between 1995 and 2015 and the closing of the gender gap. Secondly, we examine potential factors associated with their smoking behaviours. Based on the factors mentioned in existing literature and taking account of availability in the dataset, we explored the relationship to smoking prevalence of gender, survey years, perceived ease of access to cigarettes, perceived risk of smoking perceived relative wealth , parental monitoring, maternal relationship, family structure , truancy and peer smoking., also discussed the changes of the factors over time.

METHODS

Data source and sample

This study used data from the European School Survey Project on Alcohol and Other Drugs (ESPAD) Ireland. The main purpose of the survey was to collect comparable data on substance use among 15- and 16-year-old students across Europe, in order to monitor trends within and between countries, including Ireland.[19] ESPAD surveys were conducted every four years between 1995 and 2015, resulting in six waves of data from 26 countries, and 35 countries participating in 2015.

For peer review only

The sampling procedures, data collection and questionnaire used in Ireland were consistent with the international ESPAD study protocol.[19] School students born in specific calendar years were eligible and selected using stratified random sampling. Data were collected anonymously through paper-and-pencil, self-completion questionnaires administered in the classroom. After standardised cleaning procedures, the datasets were obtained from the ESPAD official database. Full accounts of the methodology of the study in each survey year could be found in the respective reports of the ESPAD project.[19 20 21]

Smoking prevalence for all six ESPAD waves is available and used for the trend analysis. The raw 1999 and 2003 survey dataset are unavailable and the 1995 dataset did not include most of the measures used in the study. Therefore, only the 2007, 2011 and 2015 studies were used to assess the associated factors of smoking. Sample characteristics are reported in Table 1**.**

Table 1 Sample characteristics of the ESPAD Ireland surveys (Between 1995 to 2015)

Years	1995	1999	2003	2007	2011	2015
Sample size	1849	2277	2407	2221	2207	1470
Male (%)	49	49	51	45	50	51
Response rate (%)	96	92	96	94	94	86

Measures

Respondents were asked how frequently they had smoked in the last 30 days, with answers ranging from “not at all” to “more than 20 cigarettes per day”. Those who answered ‘not at all’ are non-smokers and those who had smoked at least once in the last 30 days are smokers. Current or 30-day smoking prevalence rate is the proportion of smokers.

The questionnaire included items about respondents' awareness of and experience with cigarette smoking, perceived family wealth, parent monitoring, relationship with parents, family structure, truancy and peer smoking.

Students were asked how difficult it would be to get cigarettes if they wanted them and to what extent people risk harming themselves (physically or in other ways) if they smoke occasionally. The majority of students thought it would be easy to get cigarettes and about half of the students perceived a moderate/great risk from smoking occasionally.

Socioeconomic status was estimated by how well off students perceived their family to be compared to other families on 4 points from "much better off" to "less well off". Respondents were also asked whether their parents know where they spend Saturday nights (always, quite often, sometimes or usually don't know) and whether they were satisfied with their relationship with their mother. Students also listed the members of their household and around 14% of the students were from one-parent families.

Respondents were asked about truancy by reporting the number of days on which they had skipped one or more days during the last 30 days. In addition, they were asked how many of their friends smoked cigarettes.

The frequencies of responses for each predictor category are shown in Table 2. Most responses were relatively stable across the survey years but notable changes include perceived access to cigarettes, parental monitoring and peer smoking. Particularly, in 2007 and 2011, only about 12% of the students reported that none of their friends smoked, but by 2015 it had increased to 34%. The proportion of students who claimed most/all of their friends smoked had decreased from 20% in 2007 to 11% in 2015. Access to cigarettes had become more difficult across the three survey waves, with students who reported that it was difficult to obtain cigarettes increasing from 12% in 2007 to 28% in 2015. More students

For peer review only

claimed that their parents always know where they spend Saturday nights, from 48% in 2007 to 63% in 2015.

Statistical analysis

To examine the gender gap in smoking prevalence between 1995 and 2015, two-sample proportion tests were used and p-values are reported. The main analysis examined the factors associated with adolescents' smoking behaviours across the last three survey waves using multivariate logistic regression. The dependent variable was whether or not a student had smoked in the last 30 days. Independent variables included gender, survey years, and the measures listed in Table 2. The analysis was then repeated for each gender individually to detect if any factors played different roles between female and male students. All of the statistical analysis was conducted in IBM SPSS Statistics 22.

For peer review only

Table 2 Summary of the key measures

		ESPAD Year		
		2007	2011	2015
Access to cigarettes	Difficult	12.0%	16.2%	28.1%
	Easy	41.7%	41.9%	42.4%
	Don't know	46.3%	41.9%	29.5%
Risk of smoking cigarettes	No/slight risk	39.6%	43.0%	41.6%

occasionally	Moderate/great risk	58.0%	54.2%	55.8%
	Don't know	2.3%	2.8%	2.5%
Perceived family relative wealth	Much better off	15.8%	10.8%	15.8%
	Better off	32.4%	27.7%	25.8%
	About the same	46.2%	56.2%	48.6%
	Less well off	5.5%	5.3%	9.8%
Parents knowing where students spend Saturday nights	Know always	47.4%	49.6%	62.7%
	Know quite often	30.0%	30.3%	23.3%
	Know sometimes	15.4%	14.9%	8.9%
	Usually don't know	7.2%	5.3%	5.1%
Relationship with mother	Satisfied	83.4%	87.4%	87.4%
	Neither Nor	7.8%	5.0%	5.2%
	Not satisfied	8.8%	7.5%	7.3%
One-parent family	Two or more parents	87.0%	86.1%	86.0%
	One parent	13.0%	13.9%	14.0%
Skipping school in the last 30 days	None	73.6%	80.4%	80.2%
	1-4 days	21.2%	16.7%	16.1%
	5 days+	5.2%	2.9%	3.8%
Peer smoking	None	12.4%	11.9%	33.5%
	A few/some	67.6%	69.7%	55.8%
	Most/All	20.0%	18.4%	10.6%

RESULTS

Trend of adolescent smoking and the closing of gender gap

30-day smoking prevalence among boys and girls for each survey wave is shown in Table 3 and Figure 1. In 1995, female students had a 30-day smoking prevalence of 44.9%, much higher than the prevalence of male students of 36.7% (P<0.001). Along the survey years the prevalence for both genders dropped significantly, with girls achieving a greater decline. By

2015, the female and male smoking prevalence is 12.8% and 13.1%, respectively. With slightly fewer female students smoking than male students, the gender gap was closed by 2015, which is confirmed by the p-value of 0.83.

Table 3 30-day smoking prevalence (%)

Year	1995	1999	2003	2007	2011	2015
Male	36.7	32	28	19.3	18.6	13.1
Female	44.9	42	37	26.8	23.2	12.8
Total	40.9	37	33	23.4	20.9	13
P value*	<0.001	<0.001	<0.001	<0.001	0.009	0.83

* The null hypothesis is that female and male students had the same smoking prevalence.

Predictors of adolescent smoking

Table 4 presents the multivariate logistic regression results taking account of the potential factors associated with adolescent smoking. Peer smoking, perceived access to cigarettes, perceived risk of smoking, parental monitoring, truancy, maternal relationship, perceived relative wealth, family structure were all significantly associated with adolescent smoking, and some of the factors had different effects for female and male students.

Peer smoking had the strongest effect. A student with a few/some friends who smoked was 4 times more likely to smoke than a student who had no smoking friends. If most/all their friends smoked, the odds of smoking were 27 times higher for female students and 14 times higher for male students.

Students who reported a lower risk from smoking occasionally were twice more likely to smoke than those reported greater risk from smoking. For female students, those who

For peer review only - http://bmjopen.bmj.com/site/about/guidelines.xhtml

reported ‘easy to access’ cigarettes were about twice as likely to smoke as those who reported it ‘difficult’. However, for male students, there was no significant difference. Interestingly, for both genders, those who reported “don’t know” if it is easy or difficult to access cigarette, are about 3 times more likely to smoke than those who reported it as difficult.

Family appears to play an important role in adolescent smoking. Students whose parents usually do not know their whereabouts on Saturday nights are about 3 times more likely to smoke than the ones whose parents always know. For male students, the odds are even larger, at close to 5. For students who were not satisfied with their relationship with their mother, the odds of smoking are about 2 times higher than for students who were satisfied. Being from a one-parent family did not have significant effect on male smoking. However, female students from a one-parent family were about twice as likely to smoke.

Truancy was also associated with 30-day smoking, with students who skipped more days off school being more likely to smoke.

Female students were more likely to smoke than male students when controlling for the listed predictors.

Perceived family relative wealth did not matter for female students. For male students who perceived their families to be “better off” were less likely to smoke than those who answered “about the same”. Adolescents from “less well off” families were not significantly more likely to smoke than those who answered “about the same”, when controlling for the named factors. Moreover, when including both genders, students who perceived their families to be “much better off” were, however, more likely to smoke than those from average families.

Table 4 Multivariate logistic regression results

	OR (95% CI)		
	**Total**	**Male**	**Female**

For peer review only

Gender			
Male	1	**NA**	**NA**
Female	**1.50 (1.25 to 1.80)**	**NA**	**NA**
ESPAD year			
2007	1	1	1
2011	1.05 (0.85 to 1.28)	1.20 (0.89 to 1.62)	0.93 (0.70 to 1.23)
2015	0.91 (0.70 to 1.18)	1.17 (0.80 to 1.70)	0.72 (0.50 to 1.03)
Access to cigarette			
Difficult	1	1	1
Easy	**1.44 (1.01 to 2.07)**	1.12 (0.65 to 1.93)	**1.69 (1.04 to 2.74)**
Don't know	**2.88 (2.02 to 4.10)**	**2.54 (1.51 to 4.29)**	**3.04 (1.87 to 4.92)**
Perceived risk of smoking			
Great/moderate risk	1	1	1
No/slight risk	**1.90 (1.58 to 2.28)**	**1.65 (1.26 to 2.16)**	**2.16 (1.67 to 2.80)**
Don't know	0.66 (0.27 to 1.59)	0.45 (0.09 to 2.16)	0.86 (0.28 to 2.61)
Perceived family well off			
About the same	1	1	1
Much better off	**1.33 (1.02 to 1.73)**	1.15 (0.78 to 1.69)	1.42 (0.97 to 2.08)
Better off	0.84 (0.68 to 1.05)	**0.68 (0.49 to 0.93)**	1.01 (0.75 to 1.36)
Less well off	1.07 (0.74 to 1.56)	0.92 (0.52 to 1.61)	1.20 (0.73 to 1.99)
Parents know where students are on Saturday nights			
Know always	1	1	1
Know quite often	**1.46 (1.18 to 1.82)**	**1.78 (1.28 to 2.47)**	1.19 (0.88 to 1.61)
Know sometimes	**2.43 (1.89 to 3.13)**	**2.28 (1.58 to 3.29)**	**2.59 (1.81 to 3.70)**
Usually don't know	**3.24 (2.29 to 4.59)**	**4.79 (2.95 to 7.75)**	**2.04 (1.23 to 3.36)**
Relationship with mother			
Satisfied	1	1	1
Neither nor	1.33 (0.96 to 1.84)	1.36 (0.80 to 2.31)	1.34 (0.87 to 2.06)
Not satisfied	**1.82 (1.36 to 2.44)**	**1.93 (1.19 to 3.14)**	**1.81 (1.24 to 2.63)**
Family structure			
Two parents or more	1	1	1
One Parent	1.25 (0.97 to 1.61)	0.92 (0.62 to 1.38)	**1.57 (1.12 to 2.20)**
Skipping school			
None	1	1	1

1-4 days	**1.93 (1.57 to 2.38)**	**1.99 (1.47 to 2.69)**	**1.89 (1.42 to 2.52)**
5 days+	**2.80 (1.91 to 4.11)**	**2.46 (1.40 to 4.32)**	**3.46 (2.00 to 5.99)**
Friends that smoke			
None	1	1	1
A few/some	**3.77 (2.32 to 6.13)**	**3.63 (1.89 to 6.98)**	**4.23 (2.02 to 8.86)**
Most/all	**18.85 (11.39 to 31.22)**	**14.06 (7.09 to 27.87)**	**26.81 (12.51 to 57.48)**

Bold numbers indicate statistical significance at the 0.05 level.

DISCUSSION

This study confirms the decline in smoking among 15-16 year olds and the closing of the gender gap. This study also supports research showing that perceived ease of access to cigarettes, lower perceived risk of smoking, lower parental monitoring, unsatisfied family relationships, truancy, peer smoking are associated with increased adolescent smoking.

The prevalence of smoking in the past 30 days declined between 1995 and 2015, with smoking rates among girls reducing more steeply than boys, thus closing the gender gap. However, when controlling for the variables shown in Table 4, no significant change across the three survey years was found and girls had higher odds of smoking than boys did. This suggests that the factors included in the model may explain the decline in smoking prevalence; in particular those factors which have changed between data waves, namely perceived access to cigarettes, parental monitoring and particularly peer smoking.

Perceived ease of access to cigarettes decreased between 2007 and 2015. Students claiming that it was difficult to get cigarettes increased from 12% in 2007 to 28% in 2015. Several policies introduced during this period might contribute to the increase in difficulty accessing to cigarettes. In particular, the implementation of age limit law under Public Health (Tobacco) Act 2002 has officially started since April 2007. It is an offence to sell cigarettes or other tobacco products to persons aged under 18 years. In addition, since 2009 retailers are required to register with the National Tobacco Control Office. Vending machines were banned except

For peer review only

in licensed establishments. These measures followed a 2007 ban on packets containing less than 20 cigarettes and a number of substantial increases to the excise duty on tobacco products, a known effective strategy to reduce prevalence among adults and children.[22]

Parents who "know always" students' whereabouts on Saturday nights increased from 48% in 2007 to 63% in 2015. This may point to family dynamics or family culture as an important aspect of reducing adolescent smoking, although it should not be assumed that simply asking adolescents where they are going would affect their behaviour. Correlations between parental monitoring and how much caring and support they think they got may in fact be a proxy for other aspects of home and social life needing further exploration.

Peer smoking, the strongest factor for predicting adolescent smoking, has also improved between 2007 and 2015. Students with no smoking friends significantly increased from 12% to 34%, and students claiming most/all friends smoked dropped from 20% to 11%, According to the results, the odds of smoking are 27 times higher for female students who report that most/all their friends smoke than for those with no smoking friends. However, the direction of causality is not necessarily clear due to the limitation of cross-sectional study. Adolescents who smoke may seek out other smokers as friends, but equally, adolescents may imitate the behaviours of their friends, including starting to smoke.

Despite the introduction of a number of policy measures, there was little change in perceived risk associated with smoking tobacco between 2007 and 2015. Textual health warnings on cigarette packaging were introduced in 2003 and were further expanded in 2008. Further, mandatory graphic health warnings on tobacco products were introduced in 2013, so that a text-only warning occupies at least 32% of the front and a pictorial warning occupies at least 45% of the back of the pack. These measures were intended to increase awareness of the health risks associated with smoking. However, no school-based programs were developed in

For peer review only

Ireland aiming to raise the awareness of risks associated with smoking during this period. The fact that perceived risk of smoking did not increase among these respondents between 2007 and 2015 suggesting that this effect was not the mode of action of existing population-wide interventions.

There is a wealth of evidence to link adolescent smoking and low socioeconomic status, but this study has found that perceived lower relative wealth was not linked to an increase likelihood of smoking. There are some explanations for the absence of effect. First, although we meant to capture socioeconomic status by perceived family wealth compared to other student, the measure is not objective, which might raise bias on the estimation of socioeconomic effect. For example, if most of a student's friends are from very wealthy families, despite of the student's real family wealth, the student might feel he is less well off than them, which will make the measure far from the true socioeconomic status. Second, the association between low socioeconomic status and smoking may have been accounted for using other factors that were in the model. However, the reasons for the finding that 'perceived much better off family wealth' was associated with higher rates of smoking may be related to increased disposable income which is known to lessen the effects of price in adults.[23 24]

CONCLUSION

Ireland has successfully achieved a considerable decrease in adolescent smoking from 1995 to 2015. In addition, the gender gap on adolescent smoking has been closed during the period. Adolescent smoking could be further reduced through strengthening enforcement on adolescent access to cigarettes and maintaining high-intensity tobacco control media campaigns targeting adolescent to change perception of risk. Parents could contribute by

For peer review only

enhancing monitoring of offspring. It is likely that adolescent smoking will reach the Tobacco Free Ireland target of 5 percent by 2025.

For peer review only

Contributors: L C had the idea for the study, organised the team, secured the funding, helped with data collection, analysis, data interpretation, and writing. S L expanded the databases, did the final analyses and wrote a draft of paper. K T compiled the original datasets and did the initial analyses and initial draft writing. S K organised the data collection, helped with initial analysis and writing of all drafts.

Competing interests: None declared

Funding This work was supported by a grant made under an RFT for research services by the DOH for the ESPAD survey 2015; RCDHT Grant 178 supports SL.

Acknowledgements: Dr K Babineau for planning and data collection of Irish ESPAD 2015 survey, Prof Mark Morgan and Dr Zubair Kabir for advice and support.

The authors also acknowledge the ESPAD international researchers supported by EMCDDA contract CC.14.SDI.032 who compiled a common ESPAD Trend database (1995-2015)

Data sharing statement:

The data was from the European School Survey Project on Alcohol and Other Drugs (ESPAD) and various official reports available from http://www.espad.org/reports-documents.

With the 2003 data collection as a starting point it was decided that all country datasets should be merged into a common database. After that also data from 2007 and 2011 are available in separate databases. Initially, these databases were stored and maintained by the Databank Manager 'Thoroddur Bjarnson. During the 2015 wave of ESPAD, the international database was compiled and standardized by CAN (Stockholm).

Even though, since 2007, countries are obliged to deliver their national datasets to the database there are - as stated in the Database Rules - no obligations to let other researchers use the national data without permission.

In order to obtain a copy of a Database an application form has to be filled in and posted to the coordinators for further distribution to the ESPAD Application Committee. The composition of the Committee as well as restrictions around the database and its use are described and explained in the ESPAD Database Rules (Database Rules for ESPAD Researchers and Database Rules for Non–ESPAD Researchers).

When an application is approved a contract is signed before a copy of the database is delivered. Approved applications are presented in a list, which also displays the deadline of the projects.

ESPAD researchers are allowed to apply for the most recent database once the International

For peer review only

ESPAD Report has been released.

Non-ESPAD researchers are also allowed to work with ESPAD data. Access for Non-ESPAD Researchers is allowed after an embargo period determined by an Assembly:

ESPAD 2003 Database: Accessible now

ESPAD 2007 Database: Was accessible since July 1, 2013

ESPAD 2011 Database: Was accessible since July 1, 2015

ESPAD 2015 Database: At present it is only accessible to ESPAD Researchers

For peer review only

REFERENCES

[1] Breslau N, Peterson E. Smoking cessation in adolescent adults: age at initiation of cigarette smoking and other suspected influences. *Am J Public Health* 1996; 86:214-220.

[2] Everett SA, Warren CW, Sharp D, *et al*. Initiation of cigarette smoking and subsequent smoking behaviour among U.S. high school students. *Prev Med* 1999;29:327-333.

[3] Ireland Department of Health. Tobacco Free Ireland. 2013. http://health.gov.ie/wp-content/uploads/2014/03/TobaccoFreeIreland.pdf. (accessed Dec 2016).

[4] World Health Organization. WHO Framework Convention on Tobacco Control. World Health Organization, Geneva. Switzerland, 2003.

[5] Directive 2014/40/EU of the European Parliament and of the Council of 3 April 2014 on the approximation of the laws, regulations and administrative provisions of the Member States concerning the manufacture, presentation and sale of tobacco and related products and repealing Directive 2001/37/EC Text with EEA relevance.

[6] Public Health (Standardised Packaging of Tobacco) Act 2015.

[7] Carter OBJ, Phan T, Mills BW. Impact of a point-of-sale tobacco display ban on smokers' spontaneous purchases: comparisons from postpurchase interviews before and after the ban in Western Australia Tobacco Control 2015;24:e81-e86.

[8] Kuipers MAG, Beard E, Hitchman SC, *et al*. Impact on smoking of England's 2012 partial tobacco point of sale display ban: a repeated cross-sectional national study. *Tob Control* 2017;26:141-148.

[9] Stead LF, Lancaster T. Interventions for preventing tobacco sales to minors. *Cochrane Database Syst Rev*Published Online First 24 January 2005. Doi:10.1002/14651858.CD001497.pub2

[10] Roditis M, Delucchi K, Cash D, *et al.* (2016). Adolescents' perceptions of health risks, social risks, and benefits differ across tobacco products. *J Adolesc Health* 2016; 58 (5): 558-566.

[11] Rudatsikira E, Abdo A, & Muula A S. Prevalence and determinants of adolescent tobacco smoking in Addis Ababa, Ethiopia. *BMC Public Health* 2007; 7 (1): 176.

[12] Dick DM, Viken R, Purcell S, *et al*. Parental monitoring moderates the importance of genetic and environmental influences on adolescent smoking. *J Abnorm Psychol* 2007; 116: 213.

[13] Covey LS, Tam D. Depressive mood, the single-parent home, and adolescent cigarette smoking. *Am J Public Health* 1990;80:1330-1333.

[14] Ledoux S, Miller P, Choquet M, *et al*. Family structure, parent–child relationships, and alcohol and other drug use among teenagers in France and the United Kingdom. *Alcohol Alcohol* 2002;37:52-60.

[15] Simons-Morton BG. Prospective analysis of peer and parent influences on smoking initiation among early adolescents. *Prevention Science* 2002;3:275–83.

[16] Hallfors, D, Vevea, J L, Iritani, B, *et al*. Truancy, grade point average, and sexual activity: a meta-analysis of risk indicators for youth substance use. *J Sch Health* 2002;72:205–211.

[17] Amos A, Brown T, Platt S. A systematic review of the effectiveness of policies and interventions to reduce socio-economic inequalities in smoking among adolescent. *Centre for Population Health Science, University of Edinburgh* 2013. http://silne.ensp.org/wp-content/uploads/2013/09/adult-policy-report-web.docx (accessed April 2017).

[18] Brown T, Platt S, Amos A. Equity impact of interventions and policies to reduce smoking in adolescent: systematic review. *Tob Control* 2014;23(e2):e98–e105. http://doi.org/10.1136/tobaccocontrol-2013-051451

[19] The ESPAD Group. ESPAD Report 2015. Results from the European School Survey Project on Alcohol and Other Drugs. Luxembourg: Publications Office of the European Union 2016.

[20] Hibell b, Guttormsson U, Ahlström S, *et al*. The 2007 ESPAD report: substance use among students in 35 European countries. The Swedish Council for Information on Alcohol and Other Drugs 2009. http://www.drugsandalcohol.ie/11930/1/ESPAD_Report-_Full_2007.pdf (accessed Sep 2016).

For peer review only

[21] Hibell b, Guttormsson U, Ahlström S, *et al*. The 2011 ESPAD report: substance use among students in 36 European countries. The Swedish Council for Information on Alcohol and Other Drugs 2012. http://www.drugsandalcohol.ie/17644/1/The_2011_ESPAD_Report_FULL_2012-05-30.pdf (accessed Sep 2016).

[22] Currie L, Townsend J, Roux ML, et al. Policy Recommendations for Tobacco Taxation in the European Union Integrated Research Findings from the PPACTE Project. Research Institute for a Tobacco Free Society (RIFTFS) 2012. http://researchonline.lshtm.ac.uk/1249628/ (accessed Aug 2016).

[23] Alexander HM, Callcott R, Dobson AJ, et al. Cigarette smoking and drug use in schoolchildren, IV: factors associated with changes in smoking behaviour. *Int J Epidemiol* 1983;12:59–66.

[24] Stanton WR, Oei TP, Silva PA. Sociodemographic characteristics of adolescent smokers. *Int J Addict* 1994;29:913–925.

For peer review only

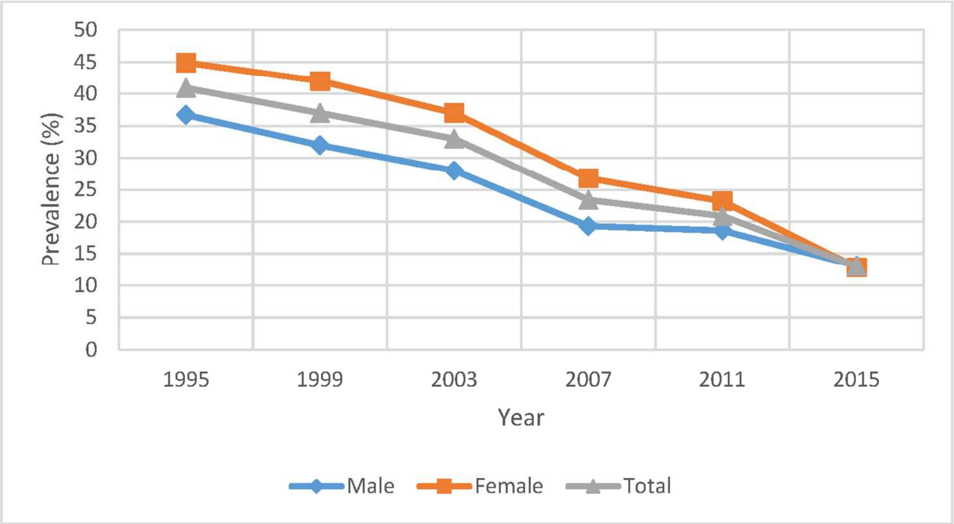

Figure 1 Trend of adolescent smoking prevalence: 1995-2015

136x77mm (300 x 300 DPI)

Appendix I: Real price per package of 20 cigarettes in Ireland, 1995-2015

Year	**1995**	**1996**	**1997**	**1998**	**1999**	**2000**	**2001**	**2002**	**2003**	**2004**	**2005**	**2006**	**2007**	**2008**	**2009**	**2010**	**2011**	**2012**	**2013**	**2014**	**2015**
Retail price	3.5	3.6	3.8	3.9	4.1	4.8	4.9	5.2	5.8	6.2	6.3	6.4	7.0	7.9	8.4	8.5	8.6	9.1	9.5	10.0	10.5
CPI (year 1995=100)	100	102	103	106	107	113	119	124	129	131	135	140	147	153	146	145	148	151	152	152	151
Real price(base year=1995)	3.5	3.6	3.6	3.7	3.8	4.2	4.1	4.2	4.5	4.7	4.6	4.6	4.8	5.2	5.7	5.8	5.8	6.0	6.2	6.6	6.9
Real price change (%)		2.6	1.9	2.4	1.6	11.6	-2.7	1.9	8.5	3.2	-1.0	-1.5	4.6	7.8	11.0	2.2	-1.4	4.9	3.3	5.3	5.3

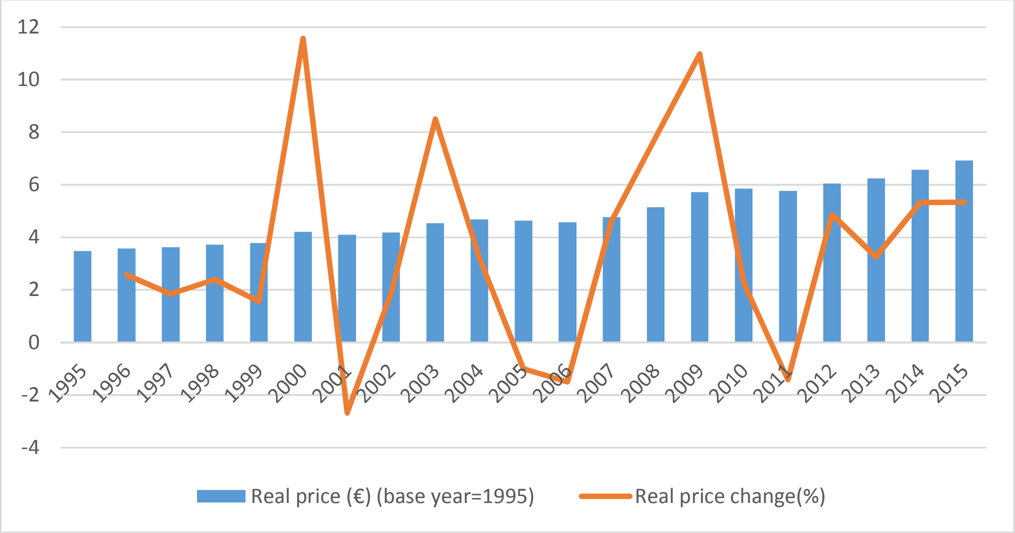

For peer review only - http://bmjopen.bmj.com/site/about/guidelines.xhtml

Appendix II: Timestamps of Tobacco Control Policies in Ireland, 1995-2015

2000
- Legislation extended the advertising ban to include print media and some forms of sponsorship.

2001
- NRT is available on prescription free of charge to medical card holders (not all polulation).

2002
- Legislation banned some forms of indirect marketing, such as mail giveaways, promotional discounts and sponsored events.
- Ireland has rotating warnings which cover 30% of the package in front and 40% of the package on the back (in accordance with EU requirements).
- Under the Public Health (Tobacco) Act 2002 it is an offence to sell cigarettes or other tobacco products to anyone under the age of 18. (Not implemented till 2007)

2003
- Ireland introduced Quiline.

2004
- Smoke-free law came into effect, which applies to all worksites, including bars and restaurants.
- Bans in advertising were extended to all forms of direct advertising in major media including billboards and more indirect advertising (ban on packages less than 20 sticks, sponsorship misleading false packaging)

2007
- Since 2 April, it is an offence to sell cigarettes or other tobacco products to persons aged under 18 years.
- A ban on packets containing less than 20 cigarettes and the sale of confectionaries that resemble cigarettes.

2008
- All products going to market after October 2008 are required to carry health warnings in both English and Irish and thus the warning size was increased to 32% of the front of the package and 45% of the rear of the package in accordance with EC Directive EC/37/2001.

2009
- Ban on point of sale display and advertising of tobacco products.
- Self-service vending machines are prohibited except in licensed premises and registered clubs and must be operated in accordance with Regulations.

2013
- Graphic warnings must be placed on any tobacco product on the market.

2014
- Since July 2014, HPRA has announced that NRT have been authorised for sale in general retail and grocery outlets, no prescription needed.

STROBE Statement—checklist of items that should be included in reports of observational studies

	Item No	Recommendation	Page
**Title and abstract**	1	(*a*) Indicate the study's design with a commonly used term in the title or the abstract	1
		(*b*) Provide in the abstract an informative and balanced summary of what was done and what was found	2
**Introduction**			
Background/rationale	2	Explain the scientific background and rationale for the investigation being reported	5
Objectives	3	State specific objectives, including any prespecified hypotheses	6
**Methods**			
Study design	4	Present key elements of study design early in the paper	7
Setting	5	Describe the setting, locations, and relevant dates, including periods of recruitment, exposure, follow-up, and data collection	7
Participants	6	(*a*) *Cohort study*—Give the eligibility criteria, and the sources and methods of selection of participants. Describe methods of follow-up<br>*Case-control study*—Give the eligibility criteria, and the sources and methods of case ascertainment and control selection. Give the rationale for the choice of cases and controls<br>*Cross-sectional study*—Give the eligibility criteria, and the sources and methods of selection of participants	7
		(*b*) *Cohort study*—For matched studies, give matching criteria and number of exposed and unexposed<br>*Case-control study*—For matched studies, give matching criteria and the number of controls per case	
Variables	7	Clearly define all outcomes, exposures, predictors, potential confounders, and effect modifiers. Give diagnostic criteria, if applicable	8
Data sources/ measurement	8*	For each variable of interest, give sources of data and details of methods of assessment (measurement). Describe comparability of assessment methods if there is more than one group	*8-9*
Bias	9	Describe any efforts to address potential sources of bias	7
Study size	10	Explain how the study size was arrived at	7
Quantitative variables	11	Explain how quantitative variables were handled in the analyses. If applicable, describe which groupings were chosen and why	8-9
Statistical methods	12	(*a*) Describe all statistical methods, including those used to control for confounding	9
		(*b*) Describe any methods used to examine subgroups and interactions	9
		(*c*) Explain how missing data were addressed	
		(*d*) *Cohort study*—If applicable, explain how loss to follow-up was addressed<br>*Case-control study*—If applicable, explain how matching of cases and controls was addressed<br>*Cross-sectional study*—If applicable, describe analytical methods taking account of sampling strategy	
		(*e*) Describe any sensitivity analyses	

For peer review only

For peer review only - http://bmjopen.bmj.com/site/about/guidelines.xhtml

**Results**			
Participants	13*	(a) Report numbers of individuals at each stage of study—eg numbers potentially eligible, examined for eligibility, confirmed eligible, included in the study, completing follow-up, and analysed	7-8
		(b) Give reasons for non-participation at each stage	
		(c) Consider use of a flow diagram	
Descriptive data	14*	(a) Give characteristics of study participants (eg demographic, clinical, social) and information on exposures and potential confounders	7-10
		(b) Indicate number of participants with missing data for each variable of interest	7
		(c) *Cohort study*—Summarise follow-up time (eg, average and total amount)	
Outcome data	15*	*Cohort study*—Report numbers of outcome events or summary measures over time	
		Case-control study—Report numbers in each exposure category, or summary measures of exposure	
		Cross-sectional study—Report numbers of outcome events or summary measures	*8-9*
Main results	16	(*a*) Give unadjusted estimates and, if applicable, confounder-adjusted estimates and their precision (eg, 95% confidence interval). Make clear which confounders were adjusted for and why they were included	11-13
		(*b*) Report category boundaries when continuous variables were categorized	
		(*c*) If relevant, consider translating estimates of relative risk into absolute risk for a meaningful time period	
Other analyses	17	Report other analyses done—eg analyses of subgroups and interactions, and sensitivity analyses	12
**Discussion**			
Key results	18	Summarise key results with reference to study objectives	14-16
Limitations	19	Discuss limitations of the study, taking into account sources of potential bias or imprecision. Discuss both direction and magnitude of any potential bias	15
Interpretation	20	Give a cautious overall interpretation of results considering objectives, limitations, multiplicity of analyses, results from similar studies, and other relevant evidence	15
Generalisability	21	Discuss the generalisability (external validity) of the study results	16
**Other information**			
Funding	22	Give the source of funding and the role of the funders for the present study and, if applicable, for the original study on which the present article is based	17

*Give information separately for cases and controls in case-control studies and, if applicable, for exposed and unexposed groups in cohort and cross-sectional studies.

Note: An Explanation and Elaboration article discusses each checklist item and gives methodological background and published examples of transparent reporting. The STROBE checklist is best used in conjunction with this article (freely available on the Web sites of PLoS Medicine at http://www.plosmedicine.org/, Annals of Internal Medicine at http://www.annals.org/, and Epidemiology at http://www.epidem.com/). Information on the STROBE Initiative is available at www.strobe-statement.org.

For peer review only

For peer review only - http://bmjopen.bmj.com/site/about/guidelines.xhtml

BMJ Open

Decline of Adolescent Smoking in Ireland 1995-2015: Trend Analysis and Associated Factors

Journal:	*BMJ Open*
Manuscript ID	bmjopen-2017-020708.R1
Article Type:	Research
Date Submitted by the Author:	23-Jan-2018
Complete List of Authors:	Li, Shasha; TobaccoFree Research Institute Ireland,<br>Keogan, Sheila; TobaccoFree Research Institute Ireland<br>Taylor, Keishia; TobaccoFree Research Institute Ireland<br>Clancy, Luke; TobaccoFree Research Institute Ireland Dublin, IE
**Primary Subject Heading**:	Smoking and tobacco
Secondary Subject Heading:	Smoking and tobacco, Epidemiology, Public health, Health policy
Keywords:	PUBLIC HEALTH, EPIDEMIOLOGY, PREVENTIVE MEDICINE

SCHOLARONE™ Manuscripts

ew only

Title: Decline of Adolescent Smoking in Ireland 1995-2015: Trend Analysis and Associated Factors

Corresponding Author: Shasha Li

Address for all authors: TobaccoFree Research Institute Ireland,

Focas Research Institute,

DIT, Kevin Street,

Dublin 8, Ireland

Email: shashali@tri.ie

Tel: +35312601966

Co-authors:

1. Sheila Keogan
2. Keishia Taylor
3. Luke Clancy

Word Count: 2808

For peer review only

ABSTRACT

Objectives

The study examines trends in smoking among Irish adolescents of 15-16 years old between 1995 and 2015 and the factors associated with their smoking behaviours between 2007 and 2015.

Methods

Data were obtained from the European School Survey Project on Alcohol and Other Drugs (ESPAD) Ireland between 1995 and 2015. To examine the gender gap, two-sample proportion tests were used. Multivariate logistic regression was performed to examine the factors associated with smoking behaviours. Dependent variable is whether a respondent is a smoker in last-30 day. Independent variables include gender, survey years, perceived ease of access to cigarettes, perceived risk of smoking, perceived relative wealth, parental monitoring, maternal relationship, family structure, truancy and peer smoking.

Results

Smoking prevalence has dropped from 41% in 1995 to 13% in 2015. The prevalence was much higher among girls than boys in 1995. The gender gap was closed by 2015. Multivariate regression results show that peer smoking, perceived access to cigarettes, perceived risks of smoking, parental monitoring, truancy, maternal relationship, perceived relative wealth and family structure were all significantly associated with adolescent smoking, and some of the factors had different effects for female and male students.

Conclusion

Ireland has successfully achieved a considerable decrease of adolescent smoking from 1995 to 2015, during which various tobacco control policies have been implemented. In addition,

For peer review only

the gender gap on adolescent smoking has been closed during the period. Adolescent smoking could be further improved through strengthening enforcement on adolescent access to cigarettes and maintaining a high-intensity tobacco control media campaign targeting adolescents. Parents could also contribute by enhancing monitoring.

Keywords: Public health; Epidemiology; Preventive medicine

For peer review only

Strengths and limitations of this study

- The data is from the best available survey on Irish adolescents from 1995-2015 using an internationally validated survey instrument, including comprehensive measurement for smoking-related factors. The data sets in different years are comparable in data collection and questions.
- The study is based on multivariable logistic regression, which controls for confounders.
- The factors associated with the changing gender gap in adolescent smoking were examined.
- Other factors potentially related to adolescent smoking were not included in the surveys, such as parental smoking.
- Limited data availability for some of the ESPAD waves are acknowledged.

For peer review only

INTRODUCTION

People who take up smoking at a younger age become more dependent and find it harder to quit than smokers who start later in their lives,[1 2] so policies designed to discourage adolescents from starting to smoke have been at the forefront of tobacco prevention in recent years. In Ireland, the Tobacco Free Ireland (TFI) report of 2013 stated that the protection of children must be prioritised in all of the initiatives outlined in the policy.[3]

The parties to the 2003 WHO Framework Convention on Tobacco Control stated their “deep concern” regarding tobacco consumption by children and adolescents, emphasising price and tax measures as effective means of reducing tobacco consumption among young people.[4] Between 1995 and 2015, the real retail price per package of 20 cigarettes in Ireland has been increased almost every year (See Appendix I). The European Tobacco Products Directive (2014/40/EU) places restrictions on the use of tobacco packaging designed to appeal to children and prohibits flavoured cigarettes and roll-your-own tobacco.[5] In Ireland, stronger legislation regarding the complete standardisation of tobacco packaging came into force in September 2017 and should reduce further the effect of tobacco advertising on adolescents.[6]

Between 1995 and 2015, a series of national level tobacco control policies were introduced (see Appendix II), although there were no school-specific tobacco control policies implemented in Ireland. For example, the implementation of age limit law under Public Health (Tobacco) Act 2002 was not fully implemented until April 2007. It is an offence to sell cigarettes or other tobacco products to persons aged under 18 years. In addition, since 2009 retailers are required to register with the National Tobacco Control Office. Vending machines were banned except in licensed establishments.

For peer review only

There has been a large volume of research conducted regarding interventions against adolescent smoking, including studies evaluating policies to restrict access and raise awareness of risk, with mixed results.[7 8 9] Some studies have examined perceptions of risk and its association with smoking and the majority reported a negative association between risk perception and smoking.[10 11] Other studies have investigated correlates in the domestic and social sphere, including associations with parental monitoring, relationships with parents, family structure, truancy from school, and peer smoking. [12 13 14 15 16] It is also established that adolescents from a lower socioeconomic background are more likely to smoke.[17 18] This study first establishes the declining trends in smoking among Irish 15 and 16 year olds between 1995 and 2015 and the closing of the gender gap. Secondly, we examine potential factors associated with their smoking behaviours using the 2007-2015 surveys. Based on the factors mentioned in existing literature and taking account of availability in the dataset, we explored the relationship to smoking prevalence of gender, survey years, perceived ease of access to cigarettes, perceived risk of smoking, perceived relative wealth, parental monitoring, maternal relationship, family structure, truancy and peer smoking, and also discussed the changes of the factors over time. We cannot assess the effects of the use of electronic(e)-cigarettes on trends using these data as we only have data on e-cigarette from the 2015 survey and they were only introduced in 2013. We have expressed our fears concerning e-cigarette and the need for close monitoring elsewhere.[19 20]

METHODS

Data source and sample

This study used data from the European School Survey Project on Alcohol and Other Drugs (ESPAD) Ireland. The main purpose of the survey was to collect comparable data on substance use among 15- and 16-year-old students across Europe, in order to monitor trends

For peer review only - http://bmjopen.bmj.com/site/about/guidelines.xhtml

within and between countries, including Ireland.[21] ESPAD surveys were conducted every four years between 1995 and 2015, resulting in six waves of data from 26 countries, and 35 countries participating in 2015.

The sampling procedures, data collection and questionnaire used in Ireland were consistent with the international ESPAD study protocol. [19] School students born in specific calendar years were eligible and selected using stratified random sampling. Data were collected anonymously through paper-and-pencil, self-completion questionnaires administered in the classroom. After standardised cleaning procedures, the datasets were obtained from the ESPAD official database. Full accounts of the methodology of the study in each survey year could be found in the respective reports of the ESPAD project. [21 22 23]

Smoking prevalence for all six ESPAD waves is available and used for the trend analysis. The raw 1999 and 2003 survey datasets are unavailable and the 1995 dataset did not include most of the measures used in the study. Therefore, only the 2007, 2011 and 2015 studies were used to assess the associated factors of smoking. Sample characteristics are reported in Table 1**.**

Table 1 Sample sizes, gender and response rates of the ESPAD Ireland surveys (1995-2015)

Years	1995	1999	2003	2007	2011	2015
Sample size	1849	2277	2407	2221	2207	1470
Male (%)	49	49	51	45	50	51
Response rate (%)	96	92	96	94	94	86

For peer review only

Measures

Respondents were asked how frequently they had smoked in the last 30 days, with answers ranging from "not at all" to "more than 20 cigarettes per day". Those who answered 'not at all' are non-smokers and those who had smoked at least once in the last 30 days are smokers. Current or 30-day smoking prevalence rate is the proportion of smokers.

The questionnaire included items about respondents' awareness of and experience with cigarette smoking, perceived family wealth, parent monitoring, relationship with parents, family structure, truancy and peer smoking.

Students were asked how difficult it would be to get cigarettes if they wanted them and to what extent people risk harming themselves (physically or in other ways) if they smoke occasionally. The majority of students thought it would be easy to get cigarettes and about half of the students perceived a moderate/great risk from smoking occasionally.

Socioeconomic status was estimated by how well off students perceived their family to be compared to other families on 4 points from "much better off" to "less well off". Respondents were also asked whether their parents know where they spend Saturday nights (always, quite often, sometimes or usually don't know) and whether they were satisfied with their relationship with their mother. Students also listed the members of their household and around 14% of the students were from one-parent families.

Respondents were asked about truancy by reporting the number of days on which they had skipped one or more days during the last 30 days. In addition, they were asked how many of their friends smoked cigarettes.

The frequencies of responses for each predictor category are shown in Table 2. Most responses changed significantly across the survey years, notably in perceived access to cigarettes, parental monitoring and peer smoking. Particularly, in 2007 and 2011, only about

For peer review only

12% of the students reported that none of their friends smoked, but by 2015 it had increased to 34%. The proportion of students who claimed most/all of their friends smoked had decreased from 20% in 2007 to 11% in 2015. Access to cigarettes had become more difficult across the three survey waves, with students who reported that it was difficult to obtain cigarettes increasing from 12% in 2007 to 28% in 2015. More students claimed that their parents always know where they spend Saturday nights, from 48% in 2007 to 63% in 2015.

Statistical analysis

To examine the gender gap in smoking prevalence between 1995 and 2015, two-sample proportion tests were used and p-values are reported. The main analysis examined the factors associated with adolescents' smoking behaviours across the last three survey waves using multivariate logistic regression. The dependent variable was whether or not a student had smoked in the last 30 days. Independent variables included gender, survey years, and the measures listed in Table 2. The analysis was then repeated for each gender individually to detect if any factors played different roles between female and male students. All of the statistical analysis was conducted in IBM SPSS Statistics 22.

For peer review only

Table 2 Summary of the results and changes in key measures associated with smoking in Irish ESPAD surveys 2007-2015

		ESPAD Year		
		2007	2011	2015
Access to cigarettes*	Difficult	12.0%	16.2%	28.1%
	Easy	41.7%	41.9%	42.4%

	Don't know	46.3%	41.9%	29.5%
Risk of smoking cigarettes occasionally	No/slight risk	39.6%	43.0%	41.6%
	Moderate/great risk	58.0%	54.2%	55.8%
	Don't know	2.3%	2.8%	2.5%
Perceived family relative wealth*	Much better off	15.8%	10.8%	15.8%
	Better off	32.4%	27.7%	25.8%
	About the same	46.2%	56.2%	48.6%
	Less well off	5.5%	5.3%	9.8%
Parents knowing where students spend Saturday nights*	Know always	47.4%	49.6%	62.7%
	Know quite often	30.0%	30.3%	23.3%
	Know sometimes	15.4%	14.9%	8.9%
	Usually don't know	7.2%	5.3%	5.1%
Relationship with mother*	Satisfied	83.4%	87.4%	87.4%
	Neither Nor	7.8%	5.0%	5.2%
	Not satisfied	8.8%	7.5%	7.3%
One-parent family	Two or more parents	87.0%	86.1%	86.0%
	One parent	13.0%	13.9%	14.0%
Skipping school in the last 30 days*	None	73.6%	80.4%	80.2%
	1-4 days	21.2%	16.7%	16.1%
	5 days+	5.2%	2.9%	3.8%
Peers smoking*	None	12.4%	11.9%	33.5%
	A few/some	67.6%	69.7%	55.8%
	Most/All	20.0%	18.4%	10.6%

* Chi Square statistically significant at 0.05 level.

RESULTS

Trend of adolescent smoking and the closing of gender gap

30-day smoking prevalence among boys and girls for each survey wave is shown in Table 3 and Figure 1. In 1995, female students had a 30-day smoking prevalence of 44.9%, much higher than the prevalence of male students of 36.7% (P<0.001). Along the survey years the

prevalence for both genders dropped significantly, with girls achieving a greater decline. By 2015, the female and male smoking prevalence is 12.8% and 13.1%, respectively. With slightly fewer female students smoking than male students, the gender gap was closed by 2015, which is confirmed by the p-value of 0.83.

Table 3 30-day smoking prevalence (%) in Irish ESPAD surveys from 1995-2015

Year	1995	1999	2003	2007	2011	2015
Male	36.7	32	28	19.3	18.6	13.1
Female	44.9	42	37	26.8	23.2	12.8
Total	40.9	37	33	23.4	20.9	13
P value*	<0.001	<0.001	<0.001	<0.001	0.009	0.83

* The null hypothesis is that female and male students had the same smoking prevalence.

Predictors of adolescent smoking

Table *4* presents the multivariate logistic regression results taking account of the potential factors associated with adolescent smoking. Peer smoking, perceived access to cigarettes, perceived risk of smoking, parental monitoring, truancy, maternal relationship, perceived relative wealth, family structure were all significantly associated with adolescent smoking, and some of the factors had different effects for female and male students.

Peer smoking had the strongest effect. A student with a few/some friends who smoked was 4 times more likely to smoke than a student who had no smoking friends. If most/all their friends smoked, the odds of smoking were 27 times higher for female students and 14 times higher for male students.

Students who reported a lower risk from smoking occasionally were twice more likely to smoke than those reported greater risk from smoking. For female students, those who reported ‘easy to access’ cigarettes were about twice as likely to smoke as those who reported it ‘difficult’. However, for male students, there was no significant difference. Interestingly, for both genders, those who reported “don’t know” if it is easy or difficult to access cigarette, are about 3 times more likely to smoke than those who reported it as difficult.

Family appears to play an important role in adolescent smoking. Students whose parents usually do not know their whereabouts on Saturday nights are about 3 times more likely to smoke than the ones whose parents always know. For male students, the odds are even larger, at close to 5 times. For students who were not satisfied with their relationship with their mother, the odds of smoking are about 2 times higher than for students who were satisfied. Being from a one-parent family did not have significant effect on male smoking. However, female students from a one-parent family were about twice as likely to smoke.

Truancy was also associated with 30-day smoking, with students who skipped more days off school being more likely to smoke.

Female students were more likely to smoke than male students when controlling for the listed predictors.

Perceived family relative wealth did not matter for female students. For male students who perceived their families to be “better off” were less likely to smoke than those who answered “about the same”. Adolescents from “less well off” families were not significantly more likely to smoke than those who answered “about the same”, when controlling for the named factors. Moreover, when including both genders, students who perceived their families to be “much better off” were, however, more likely to smoke than those from average families.

For peer review only

Table 4 Multivariate logistic regression results of various factors potentially associated with smoking from Irish ESPAD surveys 2007-2015

	OR (95% CI)		
	**Total**	**Male**	**Female**
Gender			
Male	1	**NA**	**NA**
Female	**1.50 (1.25 to 1.80)**	**NA**	**NA**
ESPAD year			
2007	1	1	1
2011	1.05 (0.85 to 1.28)	1.20 (0.89 to 1.62)	0.93 (0.70 to 1.23)
2015	0.91 (0.70 to 1.18)	1.17 (0.80 to 1.70)	0.72 (0.50 to 1.03)
Access to cigarette			
Difficult	1	1	1
Easy	**1.44 (1.01 to 2.07)**	1.12 (0.65 to 1.93)	**1.69 (1.04 to 2.74)**
Don't know	**2.88 (2.02 to 4.10)**	**2.54 (1.51 to 4.29)**	**3.04 (1.87 to 4.92)**
Perceived risk of smoking			
Great/moderate risk	1	1	1
No/slight risk	**1.90 (1.58 to 2.28)**	**1.65 (1.26 to 2.16)**	**2.16 (1.67 to 2.80)**
Don't know	0.66 (0.27 to 1.59)	0.45 (0.09 to 2.16)	0.86 (0.28 to 2.61)
Perceived family well off			
About the same	1	1	1
Much better off	**1.33 (1.02 to 1.73)**	1.15 (0.78 to 1.69)	1.42 (0.97 to 2.08)
Better off	0.84 (0.68 to 1.05)	**0.68 (0.49 to 0.93)**	1.01 (0.75 to 1.36)
Less well off	1.07 (0.74 to 1.56)	0.92 (0.52 to 1.61)	1.20 (0.73 to 1.99)
Parents know where students are on Saturday nights			
Know always	1	1	1
Know quite often	**1.46 (1.18 to 1.82)**	**1.78 (1.28 to 2.47)**	1.19 (0.88 to 1.61)
Know sometimes	**2.43 (1.89 to 3.13)**	**2.28 (1.58 to 3.29)**	**2.59 (1.81 to 3.70)**
Usually don't know	**3.24 (2.29 to 4.59)**	**4.79 (2.95 to 7.75)**	**2.04 (1.23 to 3.36)**
Relationship with mother			
Satisfied	1	1	1
Neither nor	1.33 (0.96 to 1.84)	1.36 (0.80 to 2.31)	1.34 (0.87 to 2.06)
Not satisfied	**1.82 (1.36 to 2.44)**	**1.93 (1.19 to 3.14)**	**1.81 (1.24 to 2.63)**
Family structure			

Two parents or more	1	1	1
One Parent	1.25 (0.97 to 1.61)	0.92 (0.62 to 1.38)	**1.57 (1.12 to 2.20)**
Skipping school			
None	1	1	1
1-4 days	**1.93 (1.57 to 2.38)**	**1.99 (1.47 to 2.69)**	**1.89 (1.42 to 2.52)**
5 days+	**2.80 (1.91 to 4.11)**	**2.46 (1.40 to 4.32)**	**3.46 (2.00 to 5.99)**
Friends that smoke			
None	1	1	1
A few/some	**3.77 (2.32 to 6.13)**	**3.63 (1.89 to 6.98)**	**4.23 (2.02 to 8.86)**
Most/all	**18.85 (11.39 to 31.22)**	**14.06 (7.09 to 27.87)**	**26.81 (12.51 to 57.48)**

Bold numbers indicate statistical significance at the 0.05 level.

DISCUSSION

This study confirms the decline in smoking among 15-16 year olds and the closing of the gender gap. This study also supports research showing that perceived ease of access to cigarettes, lower perceived risk of smoking, lower parental monitoring, unsatisfied family relationships, truancy, peer smoking are associated with adolescent smoking.

The prevalence of smoking in the past 30 days declined between 1995 and 2015, with smoking rates among girls reducing more steeply than boys, thus closing the gender gap. However, when controlling for the variables shown in Table *4*, no significant change across the three survey years was found and girls had higher odds of smoking than boys did. This suggests that the factors included in the model may explain the decline in smoking prevalence. In support of this we find that, most of the factors have changed significantly in a favourable direction between data waves, including perceived access to cigarettes, parental monitoring and peer smoking.

Perceived ease of access to cigarettes decreased between 2007 and 2015. Students claiming that it was difficult to get cigarettes increased from 12% in 2007 to 28% in 2015. Several policies introduced during this period might contribute to the increase in difficulty accessing

For peer review only - http://bmjopen.bmj.com/site/about/guidelines.xhtml

to cigarettes. In particular, the implementation of age limit law under Public Health (Tobacco) Act 2002 has officially started since April 2007. It is an offence to sell cigarettes or other tobacco products to persons aged under 18 years. In addition, since 2009 retailers are required to register with the National Tobacco Control Office. Vending machines were banned except in licensed establishments. These measures followed a 2007 ban on packets containing less than 20 cigarettes and a number of substantial increases to the excise duty on tobacco products, a known effective strategy to reduce prevalence among adults and children.[24]

Parents who “know always” students’ whereabouts on Saturday nights increased from 48% in 2007 to 63% in 2015. This may point to family dynamics or family culture as an important aspect of reducing adolescent smoking, although it should not be assumed that simply asking adolescents where they are going would affect their behaviour. Correlations between parental monitoring and how much caring and support they think they got may in fact be a proxy for other aspects of home and social life needing further exploration.

Peer smoking, the strongest factor for predicting adolescent smoking, has also improved between 2007 and 2015. Students with no smoking friends significantly increased from 12% to 34%, and students claiming most/all friends smoked dropped from 20% to 11%. According to the results, the odds of smoking are 27 times higher for female students who report that most/all their friends smoke than for those with no smoking friends. However, the direction of causality is not necessarily clear due to the limitation of cross-sectional study. Adolescents who smoke may seek out other smokers as friends, but equally, adolescents may imitate the behaviours of their friends, including starting to smoke. The fact that there are fewer smokers in the population may, at least partially, account for the fact that the students reported fewer friends as smokers but this would perhaps suggest a more passive or coincidental occurrence than the perceived role of peers in adolescent smoking.[25]

For peer review only

Despite the introduction of a number of policy measures, there was little change in perceived risk associated with smoking tobacco between 2007 and 2015. Textual health warnings on cigarette packaging were introduced in 2003 and were further expanded in 2008. Further, mandatory graphic health warnings on tobacco products were introduced in 2013, so that a text-only warning occupies at least 32% of the front and a pictorial warning occupies at least 45% of the back of the pack. These measures were intended to increase awareness of the health risks associated with smoking. However, no school-based programs were developed in Ireland aiming to raise the awareness of risks associated with smoking during this period. The fact that perceived risk of smoking did not increase among these respondents between 2007 and 2015 suggesting that this effect was not the mode of action of existing population-wide interventions.

There is a wealth of evidence to link adolescent smoking and low socioeconomic status, but this study has found that perceived lower relative wealth was not linked to an increase likelihood of smoking. There are some explanations for the absence of effect. First, although we meant to capture socioeconomic status by perceived family wealth compared to other student, the measure is not objective, which might raise bias on the estimation of socioeconomic effect. For example, if most of a student's friends are from very wealthy families, despite of the student's real family wealth, the student might feel he is less well off than them, which will make the measure far from the true socioeconomic status. Second, the association between low socioeconomic status and smoking may have been accounted for using other factors that were in the model. However, the reasons for the finding that 'perceived much better off family wealth' was associated with higher rates of smoking may be related to increased disposable income which is known to lessen the effects of price in adults.[26 27]

For peer review only

The closing of the gap between females and males is not fully explained by the changes in the variables examined alone and may be due to a differential effect of the changes in legislation which will be examined in other data sources.

CONCLUSION

Ireland has successfully achieved a considerable decrease in adolescent smoking from 1995 to 2015. In addition, the gender gap on adolescent smoking has been closed during the period. Decreased access to cigarettes has been associated with decreased smoking and the results show that better implementation of legislation is possible and should lead to further declines in the prevalence of smoking. The perception of the risks of smoking however has not increased suggesting that targeted high-intensity tobacco control media campaigns may help and should be implemented.

The results also suggest that parents could contribute to further declines in smoking by enhancing monitoring of offspring. It is likely that adolescent smoking will reach the Tobacco Free Ireland target of 5 percent by 2025 given the rate of decline in this age group.

For peer review only

Contributors: L C had the idea for the study, organised the team, secured the funding, helped with data collection, analysis, data interpretation, and writing. S L expanded the databases, did the final analyses and wrote a draft of paper. K T compiled the original datasets and did the initial analyses and initial draft writing. S K organised the data collection, helped with initial analysis and writing of all drafts.

Competing interests: None declared

Funding This work was supported by a grant made under an RFT for research services by the DOH for the ESPAD survey 2015; RCDHT Grant 178 supports SL.

Acknowledgements: Dr K Babineau for planning and data collection of Irish ESPAD 2015 survey, Prof Mark Morgan and Dr Zubair Kabir for advice and support.

The authors also acknowledge the ESPAD international researchers supported by EMCDDA contract CC.14.SDI.032 who compiled a common ESPAD Trend database (1995-2015)

Data sharing statement:

The data was from the European School Survey Project on Alcohol and Other Drugs (ESPAD) and various official reports available from http://www.espad.org/reports-documents.

With the 2003 data collection as a starting point it was decided that all country datasets should be merged into a common database. After that also data from 2007 and 2011 are available in separate databases. Initially, these databases were stored and maintained by the Databank Manager 'Thoroddur Bjarnson. During the 2015 wave of ESPAD, the international database was compiled and standardized by CAN (Stockholm).

Even though, since 2007, countries are obliged to deliver their national datasets to the database there are - as stated in the Database Rules - no obligations to let other researchers use the national data without permission.

In order to obtain a copy of a Database an application form has to be filled in and posted to the coordinators for further distribution to the ESPAD Application Committee. The composition of the Committee as well as restrictions around the database and its use are described and explained in the ESPAD Database Rules (Database Rules for ESPAD Researchers and Database Rules for Non–ESPAD Researchers).

For peer review only

When an application is approved a contract is signed before a copy of the database is delivered. Approved applications are presented in a list, which also displays the deadline of the projects.

ESPAD researchers are allowed to apply for the most recent database once the International ESPAD Report has been released.

Non-ESPAD researchers are also allowed to work with ESPAD data. Access for Non-ESPAD Researchers is allowed after an embargo period determined by an Assembly:

ESPAD 2003 Database: Accessible now

ESPAD 2007 Database: Was accessible since July 1, 2013

ESPAD 2011 Database: Was accessible since July 1, 2015

ESPAD 2015 Database: At present it is only accessible to ESPAD Researchers

For peer review only

REFERENCES

[1] Breslau N, Peterson E. Smoking cessation in adolescent adults: age at initiation of cigarette smoking and other suspected influences. *Am J Public Health* 1996;86:214-220.

[2] Everett SA, Warren CW, Sharp D, *et al*. Initiation of cigarette smoking and subsequent smoking behaviour among U.S. high school students. *Prev Med* 1999;29:327-333.

[3] Ireland Department of Health. Tobacco Free Ireland. 2013. http://health.gov.ie/wp-content/uploads/2014/03/TobaccoFreeIreland.pdf. (accessed Dec 2016).

[4] World Health Organization. WHO Framework Convention on Tobacco Control. World Health Organization, Geneva. Switzerland, 2003.

[5] Directive 2014/40/EU of the European Parliament and of the Council of 3 April 2014 on the approximation of the laws, regulations and administrative provisions of the Member States concerning the manufacture, presentation and sale of tobacco and related products and repealing Directive 2001/37/EC Text with EEA relevance.

[6] Public Health (Standardised Packaging of Tobacco) Act 2015.

[7] Carter OBJ, Phan T, Mills BW. Impact of a point-of-sale tobacco display ban on smokers' spontaneous purchases: comparisons from postpurchase interviews before and after the ban in Western Australia Tobacco Control 2015;24:e81-e86.

[8] Kuipers MAG, Beard E, Hitchman SC, *et al*. Impact on smoking of England's 2012 partial tobacco point of sale display ban: a repeated cross-sectional national study. *Tob Control* 2017;26:141-148.

[9] Stead LF, Lancaster T. Interventions for preventing tobacco sales to minors. *Cochrane Database Syst Rev*Published Online First 24 January 2005. Doi:10.1002/14651858.CD001497.pub2

[10] Roditis M, Delucchi K, Cash D, *et al.* (2016). Adolescents' perceptions of health risks, social risks, and benefits differ across tobacco products. *J Adolesc Health* 2016; 58 (5): 558-566.

For peer review only

[11] Rudatsikira E, Abdo A, & Muula A S. Prevalence and determinants of adolescent tobacco smoking in Addis Ababa, Ethiopia. *BMC Public Health* 2007; 7 (1): 176.

[12] Dick DM, Viken R, Purcell S, *et al*. Parental monitoring moderates the importance of genetic and environmental influences on adolescent smoking. *J Abnorm Psychol* 2007; 116: 213.

[13] Covey LS, Tam D. Depressive mood, the single-parent home, and adolescent cigarette smoking. *Am J Public Health* 1990;80:1330-1333.

[14] Ledoux S, Miller P, Choquet M, *et al*. Family structure, parent–child relationships, and alcohol and other drug use among teenagers in France and the United Kingdom. *Alcohol Alcohol* 2002;37:52-60.

[15] Simons-Morton BG. Prospective analysis of peer and parent influences on smoking initiation among early adolescents. *Prevention Science* 2002;3:275–83.

[16] Hallfors, D, Vevea, J L, Iritani, B, *et al*. Truancy, grade point average, and sexual activity: a meta-analysis of risk indicators for youth substance use. *J Sch Health* 2002;72:205–211.

[17] Amos A, Brown T, Platt S. A systematic review of the effectiveness of policies and interventions to reduce socio-economic inequalities in smoking among adolescent. *Centre for Population Health Science, University of Edinburgh* 2013. http://silne.ensp.org/wp-content/uploads/2013/09/adult-policy-report-web.docx (accessed April 2017).

[18] Brown T, Platt S, Amos A. Equity impact of interventions and policies to reduce smoking in adolescent: systematic review. *Tob Control* 2014;23(e2):e98–e105. http://doi.org/10.1136/tobaccocontrol-2013-051451

[19] Babineau K, Taylor K, Clancy L. Electronic cigarette use among Irish Youth: a cross sectional study of prevalence and associated factors. *PLoS ONE* 2015;10(5):e0126419.

For peer review only

[20] Clancy L, Babineau K. E-cigarettes: effective cessation tools or public health threat? *QJM* 2016;109(2):79-81.

[21] The ESPAD Group. ESPAD Report 2015. Results from the European School Survey Project on Alcohol and Other Drugs. Luxembourg: Publications Office of the European Union 2016.

[22] Hibell b, Guttormsson U, Ahlström S, *et al*. The 2007 ESPAD report: substance use among students in 35 European countries. The Swedish Council for Information on Alcohol and Other Drugs 2009. http://www.drugsandalcohol.ie/11930/1/ESPAD_Report-_Full_2007.pdf (accessed Sep 2016).

[23] Hibell b, Guttormsson U, Ahlström S, *et al*. The 2011 ESPAD report: substance use among students in 36 European countries. The Swedish Council for Information on Alcohol and Other Drugs 2012. http://www.drugsandalcohol.ie/17644/1/The_2011_ESPAD_Report_FULL_2012-05-30.pdf (accessed Sep 2016).

[24] Currie L, Townsend J, Roux ML, et al. Policy Recommendations for Tobacco Taxation in the European Union Integrated Research Findings from the PPACTE Project. Research Institute for a Tobacco Free Society (RIFTFS) 2012. http://researchonline.lshtm.ac.uk/1249628/ (accessed Aug 2016).

[25] Stewart-Knox BJ, Sittlington J, Rugkåsa J, et al. Smoking and peer groups: results from a longitudinal qualitative study of young people in Northern Ireland. *Br J Soc Psychol* 2005;44(3):397-414.

[26] Alexander HM, Callcott R, Dobson AJ, et al. Cigarette smoking and drug use in schoolchildren, IV: factors associated with changes in smoking behaviour. *Int J Epidemiol* 1983;12:59–66.

For peer review only

[27] Stanton WR, Oei TP, Silva PA. Sociodemographic characteristics of adolescent smokers. *Int J Addict* 1994;29:913–925.

Figure 1 Trend of adolescent smoking prevalence: 1995-2015

For peer review only

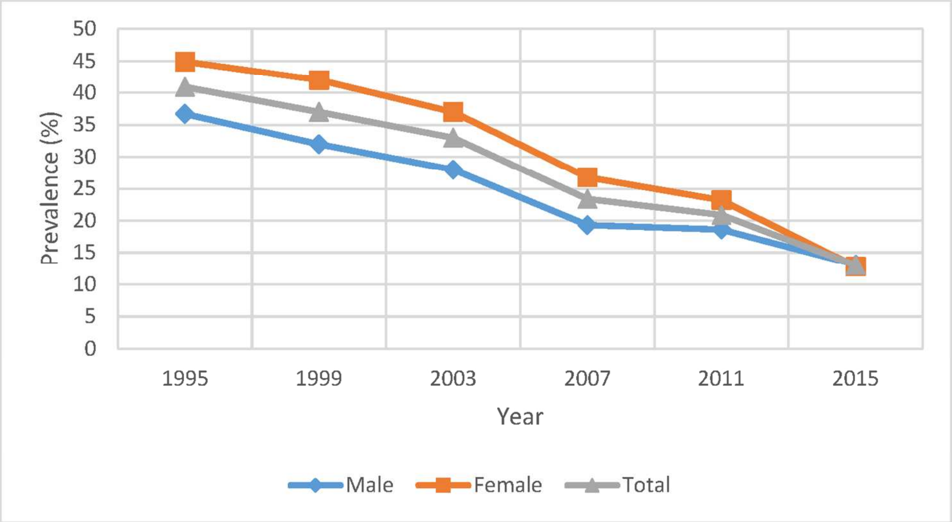

Figure 1 Trend of adolescent smoking prevalence: 1995-2015

136x77mm (300 x 300 DPI)

For peer review only

Appendix I: Real price per package of 20 cigarettes in Ireland, 1995-2015

Year	1995	1996	1997	1998	1999	2000	2001	2002	2003	2004	2005	2006	2007	2008	2009	2010	2011	2012	2013	2014	2015
Retail price	3.5	3.6	3.8	3.9	4.1	4.8	4.9	5.2	5.8	6.2	6.3	6.4	7.0	7.9	8.4	8.5	8.6	9.1	9.5	10.0	10.5
CPI (year 1995=100)	100	102	103	106	107	113	119	124	129	131	135	140	147	153	146	145	148	151	152	152	151
Real price(base year=1995)	3.5	3.6	3.6	3.7	3.8	4.2	4.1	4.2	4.5	4.7	4.6	4.6	4.8	5.2	5.7	5.8	5.8	6.0	6.2	6.6	6.9
Real price change (%)		2.6	1.9	2.4	1.6	11.6	-2.7	1.9	8.5	3.2	-1.0	-1.5	4.6	7.8	11.0	2.2	-1.4	4.9	3.3	5.3	5.3

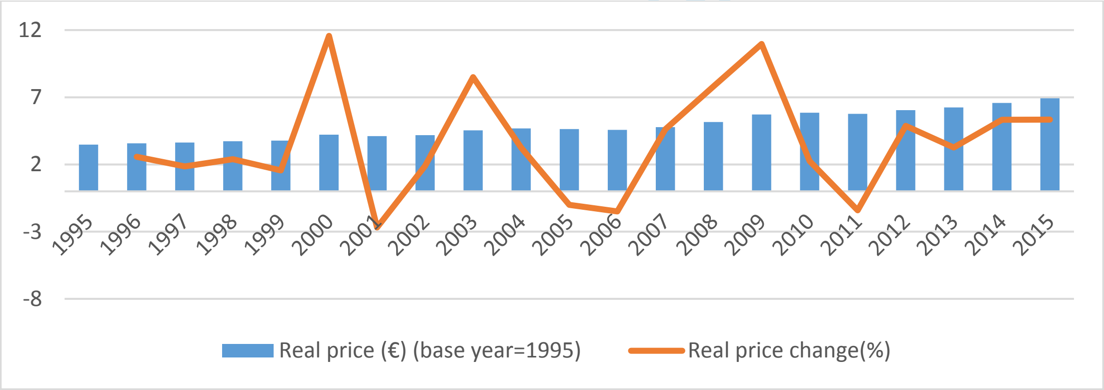

For peer review only - http://bmjopen.bmj.com/site/about/guidelines.xhtml

Appendix II: Timestamps of Tobacco Control Policies in Ireland, 1995-2015

2000
- Legislation extended the advertising ban to include print media and some forms of sponsorship.

2001
- NRT is available on prescription free of charge to medical card holders (not all polulation).

2002
- Legislation banned some forms of indirect marketing, such as mail giveaways, promotional discounts and sponsored events.
- Ireland has rotating warnings which cover 30% of the package in front and 40% of the package on the back (in accordance with EU requirements).
- Under the Public Health (Tobacco) Act 2002 it is an offence to sell cigarettes or other tobacco products to anyone under the age of 18. (Not implemented till 2007)

2003
- Ireland introduced Quiline.

2004
- Smoke-free law came into effect, which applies to all worksites, including bars and restaurants.
- Bans in advertising were extended to all forms of direct advertising in major media including billboards and more indirect advertising (ban on packages less than 20 sticks, sponsorship misleading false packaging)

2007
- Since 2 April, it is an offence to sell cigarettes or other tobacco products to persons aged under 18 years.
- A ban on packets containing less than 20 cigarettes and the sale of confectionaries that resemble cigarettes.

2008
- All products going to market after October 2008 are required to carry health warnings in both English and Irish and thus the warning size was increased to 32% of the front of the package and 45% of the rear of the package in accordance with EC Directive EC/37/2001.

2009
- Ban on point of sale display and advertising of tobacco products.
- Self-service vending machines are prohibited except in licensed premises and registered clubs and must be operated in accordance with Regulations.

2013
- Graphic warnings must be placed on any tobacco product on the market.

2014
- Since July 2014, HPRA has announced that NRT have been authorised for sale in general retail and grocery outlets, no prescription needed.

STROBE Statement—checklist of items that should be included in reports of observational studies

	Item No	Recommendation	Page
**Title and abstract**	1	(*a*) Indicate the study's design with a commonly used term in the title or the abstract	1
		(*b*) Provide in the abstract an informative and balanced summary of what was done and what was found	2
**Introduction**			
Background/rationale	2	Explain the scientific background and rationale for the investigation being reported	5
Objectives	3	State specific objectives, including any prespecified hypotheses	6
**Methods**			
Study design	4	Present key elements of study design early in the paper	7
Setting	5	Describe the setting, locations, and relevant dates, including periods of recruitment, exposure, follow-up, and data collection	7
Participants	6	(*a*) *Cohort study*—Give the eligibility criteria, and the sources and methods of selection of participants. Describe methods of follow-up<br>*Case-control study*—Give the eligibility criteria, and the sources and methods of case ascertainment and control selection. Give the rationale for the choice of cases and controls<br>*Cross-sectional study*—Give the eligibility criteria, and the sources and methods of selection of participants	7
		(*b*) *Cohort study*—For matched studies, give matching criteria and number of exposed and unexposed<br>*Case-control study*—For matched studies, give matching criteria and the number of controls per case	
Variables	7	Clearly define all outcomes, exposures, predictors, potential confounders, and effect modifiers. Give diagnostic criteria, if applicable	8
Data sources/ measurement	8*	For each variable of interest, give sources of data and details of methods of assessment (measurement). Describe comparability of assessment methods if there is more than one group	*8-9*
Bias	9	Describe any efforts to address potential sources of bias	7
Study size	10	Explain how the study size was arrived at	7
Quantitative variables	11	Explain how quantitative variables were handled in the analyses. If applicable, describe which groupings were chosen and why	8-9
Statistical methods	12	(*a*) Describe all statistical methods, including those used to control for confounding	9
		(*b*) Describe any methods used to examine subgroups and interactions	9
		(*c*) Explain how missing data were addressed	
		(*d*) *Cohort study*—If applicable, explain how loss to follow-up was addressed<br>*Case-control study*—If applicable, explain how matching of cases and controls was addressed<br>*Cross-sectional study*—If applicable, describe analytical methods taking account of sampling strategy	
		(*e*) Describe any sensitivity analyses	

For peer review only

**Results**			
Participants	13*	(a) Report numbers of individuals at each stage of study—eg numbers potentially eligible, examined for eligibility, confirmed eligible, included in the study, completing follow-up, and analysed	7-8
		(b) Give reasons for non-participation at each stage	
		(c) Consider use of a flow diagram	
Descriptive data	14*	(a) Give characteristics of study participants (eg demographic, clinical, social) and information on exposures and potential confounders	7-10
		(b) Indicate number of participants with missing data for each variable of interest	7
		(c) *Cohort study*—Summarise follow-up time (eg, average and total amount)	
Outcome data	15*	*Cohort study*—Report numbers of outcome events or summary measures over time	
		Case-control study—Report numbers in each exposure category, or summary measures of exposure	
		Cross-sectional study—Report numbers of outcome events or summary measures	*8-9*
Main results	16	(*a*) Give unadjusted estimates and, if applicable, confounder-adjusted estimates and their precision (eg, 95% confidence interval). Make clear which confounders were adjusted for and why they were included	11-13
		(*b*) Report category boundaries when continuous variables were categorized	
		(*c*) If relevant, consider translating estimates of relative risk into absolute risk for a meaningful time period	
Other analyses	17	Report other analyses done—eg analyses of subgroups and interactions, and sensitivity analyses	12
**Discussion**			
Key results	18	Summarise key results with reference to study objectives	14-16
Limitations	19	Discuss limitations of the study, taking into account sources of potential bias or imprecision. Discuss both direction and magnitude of any potential bias	15
Interpretation	20	Give a cautious overall interpretation of results considering objectives, limitations, multiplicity of analyses, results from similar studies, and other relevant evidence	15
Generalisability	21	Discuss the generalisability (external validity) of the study results	16
**Other information**			
Funding	22	Give the source of funding and the role of the funders for the present study and, if applicable, for the original study on which the present article is based	17

*Give information separately for cases and controls in case-control studies and, if applicable, for exposed and unexposed groups in cohort and cross-sectional studies.

Note: An Explanation and Elaboration article discusses each checklist item and gives methodological background and published examples of transparent reporting. The STROBE checklist is best used in conjunction with this article (freely available on the Web sites of PLoS Medicine at http://www.plosmedicine.org/, Annals of Internal Medicine at http://www.annals.org/, and Epidemiology at http://www.epidem.com/). Information on the STROBE Initiative is available at www.strobe-statement.org.

For peer review only
